# Trends in childhood routine immunisation and maternal health service access and utilisation in Sokoto State, Nigeria. A quantitative survey

**DOI:** 10.1038/s41598-025-30637-7

**Published:** 2025-12-03

**Authors:** Clovis Nchinjoh Sangwe, Wilfred Njabulo Nunu, Kelebogile Leah Manjinja, Sibusiso Frank Nkosi

**Affiliations:** 1 Division of Public Health, Faculty of Health Sciences, University of the Free State, Bloemfontein, South Africa; 2 Department of Environmental Health, School of Public Health, Faculty of Health Sciences, University of Botswana, Gaborone, Botswana

**Keywords:** Routine immunisation, Maternal health, COVID-19, Pandemic resilience framework, Diseases, Health care, Medical research

## Abstract

Sokoto State, Nigeria, continues to report some of the lowest maternal, neonatal, and child health (MNCH) indicators nationally. The COVID-19 pandemic further disrupted service delivery in Nigeria, yet its long-term impact on routine immunisation (RI) and maternal health services at the sub-national level in fragile and resource-constrained settings remains underexplored. We conducted a retrospective cohort study using secondary administrative data from 2015 to 2023 to examine the trends in the uptake of the first and third doses of the diphtheria-tetanus-pertussis containing vaccines (DTP-1 and DTP-3), and facility-based deliveries. Dynamic time-series regression models were applied to assess the influence of the COVID-19 period and contextual factors, including climate change, settlement type, and health budgets. DTP-1 coverage and proportion of facility deliveries improved over time. There was a significant increase in facility deliveries during the COVID-19 pandemic (*p* = 0.019), while DTP-1 coverage declined modestly during the pandemic (*p* = 0.030). DTP-3 showed no significant change (*p* = 0.078). Urban residence was positively associated with facility delivery. Lagged dependent variables were strongly significant across the models, indicating path dependency in service utilisation. These findings highlight both resilience and vulnerability within Sokoto’s healthcare system. While maternal services were adapted during the pandemic, immunisation continuity remained fragile. These insights could inform the development of a pandemic-resilient framework for MNCH services in similar settings.

## Introduction

Maternal, neonatal, and child health (MNCH) services, including routine immunisation (RI), antenatal care (ANC), and skilled delivery, are essential for reducing preventable morbidity and mortality among women and children^1,2^. Global health initiatives have contributed to notable improvements in immunisation coverage and maternal survival; however, progress remains uneven, particularly in low-resource settings^3,4^. Sub-Saharan Africa (SSA) continues to bear the highest burden of maternal and child deaths, largely because of persistent inequities in access to and utilisation of essential health services^3,4^.

Childhood immunisation has been instrumental in reducing under-five mortality, contributing to the eradication of smallpox and certification of the African region as wild polio-free^5^. However, disparities in vaccine coverage have persisted^6^. In 2021, millions of children in Africa missed critical vaccinations, with coverage rates for DTP1 and DTP3 being significantly below global averages. Maternal health indicators reveal similar gaps^7^. Despite the WHO recommendations for a minimum of eight ANC visits, only a minority of pregnant women in Western and Central Africa receive four or more visits^8^. These disparities are exacerbated by urban-rural and wealth-related inequalities, with SSA exhibiting the widest gaps in skilled birth attendance and ANC coverage^9^.

The COVID-19 pandemic has further strained global health systems, with SSA experiencing disproportionate disruptions due to limited infrastructure, inequitable resource distribution, and weak operational capacity^10–12^. In Nigeria, the pandemic led to marked declines in ANC visits, skilled deliveries, and RI uptake, particularly in the northern states^13–17^. Misinformation and inadequate communication during the COVID-19 vaccine rollout intensified vaccine hesitancy, which spilled over to routine childhood immunisation, threatening decades of progress^18,19^.

Sokoto State, located in Nigeria’s northwest geopolitical zone, exemplifies these challenges. With over 1.5 million women of childbearing age and nearly 750,000 children under five, the state consistently reports some of the lowest MNCH service coverage rates nationally^20,21^. In 2022, ANC coverage stood at 30.5%, and childhood vaccination coverage was only 11.5%, both significantly below the national and regional benchmarks^21^. The majority of deliveries occur at home, often without skilled assistance, reflecting entrenched barriers to accessing formal healthcare^20^. These health indicators reflect critical challenges, with an under-five mortality rate (U5MR) of 202 per 1,000 live births and a maternal mortality ratio (MMR) of 985 per 100,000 live births, figures that exceed the 2030 Sustainable Development Goal (SDG) targets by factors of eight and fourteen, respectively^21^.

While national surveys and global reports have documented broad trends in MNCH service delivery, there is a lack of granular, longitudinal evidence examining the temporal dynamics and drivers of change in RI and maternal health indicators at the subnational level in Nigeria^3,4,21,22^. This gap is particularly critical in Sokoto State, where health system constraints, socio-cultural barriers, insecurity, and geographic inequities converge to produce some of the country’s poorest MNCH outcomes^21^. Moreover, although the impact of the COVID-19 pandemic on health service delivery has been widely acknowledged, few studies have systematically quantified its effects on RI and maternal health service utilisation over time in high-burden, resource-constrained settings^13,14,23^. The absence of a pandemic-resilient framework tailored to Sokoto’s context underscores the need for empirical evidence to guide policy and strategic planning. The aim of the study was therefore to analyse the trends in childhood RI and maternal health service access and utilisation in Sokoto State from 2015 to 2023 as detailed in Table 1. This included exploring trends in RI and maternal health service performance indicators, and identifying the factors associated with significant changes in the indicators. The findings will contribute to the development of context-specific, evidence-based strategies to strengthen health system resilience and improve MNCH outcomes in Sokoto and similar settings.

**Table 1:** Summary of Objectives, data Sources, and analysis Plan.

Objectives	Data Source(s)	Variables	Analysis
1. To analyse the RI and maternal performance indicator trends in Sokoto State from 2015 to 2023.	DHIS-2	DTP-1 coverage, DTP-3 coverage, proportion of facility deliveries	Descriptive trend analysis
2. To identify the factors associated with significant changes in RI and maternal performance indicators between 2015 and 2023.	DHIS-2, NIMET data, Desk review	**Dependent variables**: DTP-1 coverage, DTP-3 coverage, proportion of facility deliveries, **Independent variables**: COVID-19 pandemic period (Yes/No) **Potential confounders**: minimum temperature, thunderstorms, rain days, annual maternal health budget, annual health budget, and settlement types.	Dynamic time series regression analysis

Nimet - Nigeria Meteorological Agency, DHIS-2 - District Health Information System version 2, DPT-1 and DTP-3–1st and 3rd dose of diphtheria-tetanus-pertussis containing vaccine, COVID-19 - Coronavirus Disease 2019

## Methodology

### Geographical setting

Sokoto State is situated in northwestern Nigeria, sharing borders with Kebbi State to the west, Zamfara State to the south, Katsina State to the east, and the Republic of Niger to the north^24^. Geographically, the state lies within Nigeria’s Sudan Savannah zone, a region characterised by semi-arid climatic conditions and sparse vegetation typical of the Sahel belt^24^. According to the most recent estimates from the National Population Commission (NPC), Sokoto has a population of approximately 4.7 million, ranking it the 17th most populous state in the country^25^.

Administratively, the state comprises 23 Local Government Areas (LGAs), each hosting an average of ten primary healthcare centres^26^. Of these LGAs, five were classified as urban, while the remaining 18 were predominantly rural^27^. For health service planning and coordination, the state is subdivided into three health zones: North, East, and South^26^. Established in 1976 following the division of the former North-Western state, Sokoto is largely inhabited by Hausa and Fulani ethnic groups, with Islam being the dominant religion^28^. As reported by the National Bureau of Statistics, 87.7% of the population live below the poverty line, making Sokoto the poorest state in Nigeria^27^.

### Study design

This study employed an analytical retrospective cohort design, drawing on secondary data related to RI and maternal health performance indicators in Sokoto State, between 2015 and 2023. Aggregated secondary data at the LGA and state levels, sourced from publicly available databases such as DHIS-2, NIMET and official reports, were used. No individual-level data were collected or analysed, and no human subjects were directly involved and so informed consent was not applicable as approved by the Health Science Research Ethics Committee of the Faculty of Health Sciences at the University of the Free State, South Africa (UFS-HSD2025/0102/2705 on the 28th May 2025), and the Sokoto State Health Research Ethics Committee (SMH/1580/V. IV. on 19th December 2024).

This design enabled the examination of temporal patterns in the coverage of the first and third doses of the Diphtheria-Tetanus-Pertussis-containing vaccine (DTP-1 and DTP-3) and the proportion of facility-based deliveries over time, thereby facilitating the identification of long-term trends and associations between key variables^29–31^. Existing administrative datasets, including records indicating whether COVID-19 cases were reported, were utilised to assess the influence of these exposures on RI and maternal health indicators^32^. This approach offers a cost-effective and time-efficient means of exploring associations by linking exposures (COVID-19 pandemic) with outcomes (DTP-1 and DTP-3 coverage and facility delivery rates)^33^. It also allows for the establishment of temporal relationships and potential associations between variables^33^. To strengthen the validity and reliability of the findings, potential confounding factors such as average temperature, annual health budget, settlement type, rain days, thunderstorms, and healthcare infrastructure were incorporated into the analysis^33^.

### Quantitative data collection

The study drew on secondary data sources related to RI and maternal health performance indicators, COVID-19 pandemic-related exposures, and relevant confounding variables in Sokoto State. Data were obtained from administrative records available through the District Health Information System (DHIS-2), Nigeria Meteorological Agency (NIMET), and a review of relevant reports spanning the period from 2015 to 2023^32^. This timeframe was selected to enable a comprehensive analysis of the long-term trends^31^ in DTP-1 and DTP-3 coverage and the proportion of facility-based deliveries. Similar studies employing retrospective cohort designs have examined data across an extended period to identify fluctuations in healthcare utilisation patterns and the influence of contextual factors such as health policies and disease outbreaks (for example, COVID-19)^29–31^.

This study utilised secondary data extracted from the DHIS2, which is part of Nigeria’s Health Management Information System (HMIS)^34^. The data were obtained using a standardised checklist aligned with HMIS reporting formats to ensure consistency across variables and facilitate comparative analysis^34^. The HMIS employs nationally standardised data collection tools and procedures, which have been validated by public health and epidemiology experts^35^. Content validity is ensured through the national standardisation process, which aligns data elements with key health system performance indicators. Reliability is supported by routine data quality assessments and periodic reviews conducted by health authorities^35^. Although the authors did not conduct primary data collection, the study relied on these established systems to ensure the integrity of the dataset.

### Data management and analysis

The dataset on RI and maternal health performance indicators, including DTP-1 coverage, DTP-3 coverage, and the proportion of facility deliveries, were exported from Microsoft Excel 2013 into R Statistical Software (v4.1.2; R Core Team, 2021) for statistical analysis.

Continuous variables were adjusted by weighting them against the Sokoto State’s DHIS-2 data completeness for 2023. This adjustment ensures that observed values are scaled relative to the completeness of Sokoto State’s DHIS-2 data in 2023, which served as the reference benchmark. This adjustment was calculated using the following formula^36^:

$$\begin{aligned} & Adjusted~\text{var} iable~value\left( {e.g.~~adjusted~DTP - 1~\text{cov} erage} \right) \\ & = \left( {{{2023~Sokoto~State~data~completeness} \mathord{\left/ {\vphantom {{2023~Sokoto~State~data~completeness} {data~completeness~for~the~\text{var} iable}}} \right. \kern-\nulldelimiterspace} {data~completeness~for~the~\text{var} iable}}} \right) \\ & \times ~Original~\text{var} iable~value \\ \end{aligned}$$

Where:

- 2023 Sokoto State data completeness is a reference completeness value (e.g. 95.6% completeness for 2023 DTP-1 indicator).
- Data Completeness for the Variable is the completeness rate for the specific variable in a given year.
- Original Variable Value is the unadjusted value from DHIS-2.

To illustrate, consider the DTP-1 coverage in January 2016, which was 70.8%, with a corresponding data completeness of 81.5%. Using the reference completeness of 95.6% for 2023, the adjusted value is calculated as:

$$\:Adjusted\:January\:2016\:DTP-1\:coverage=\frac{95.6}{81.5}\:X\:70.8\%=83\%$$

Some annual coverage values exceed the theoretical maximum of 100%. These figures are recognised as artefacts of administrative data reporting and may result from factors such as inaccuracies in population denominators, cross-border service utilisation, or duplicate reporting. While these values were retained for analytical consistency, they should not be interpreted as literal coverage levels. Rather, they reflect potential data quality issues common in routine health information systems and should be viewed as indicative of reporting trends rather than precise service uptake.

Continuous variables were summarised using measures of central tendency, while categorical variables were presented as percentages through cross-tabulations. A descriptive trend analysis was conducted to examine long-term patterns across the study period. Annual trends provided insight into year-on-year changes, whereas monthly trends highlighted short-term fluctuations during specific periods of interest.

The dataset included both dependent variables (DTP-1 coverage, DTP-3 coverage, and proportion of facility deliveries) and independent variables (COVID-19 reporting status), along with potential confounders, such as average temperature, annual health budget, settlement type, rain days, and thunderstorms. These variables were analysed using dynamic time-series regression^37^, which allowed for the incorporation of lagged effects and temporal dependencies^37^. This is particularly important in health systems research, where the impact of interventions or disruptions such as outbreaks, campaigns, or policy changes may not be immediate but delayed^36^.

Unlike traditional time-series models that assume stationary relationships, dynamic time-series regression models are better suited for capturing the evolving and complex interactions among multiple independent variables and their collective influence on outcome indicators^37^. This approach also enabled the modelling of nonlinear relationships and time-varying coefficients, which are common in public health contexts where interventions and external factors shift over time^37^. This flexibility provided a more nuanced understanding of how variables interacted with and influenced RI and maternal health outcomes in Sokoto State.

To construct the model, preliminary time-series analyses were conducted for the time-dependent explanatory variable (COVID-19 reporting status) to assess its association with RI and maternal health indicators (DTP-1 and DTP-3 coverage and facility delivery rates)^37^. Using the same approach, potential confounders, including climatic, infrastructural, and policy-related factors, were incorporated to strengthen the validity and reliability of the findings by accounting for variables that may independently influence service uptake^38^.

Explanatory variables p-value < 0.2 from univariate analyses were retained for multivariate modelling. Given the complexity of RI and maternal health access and utilisation, this approach allowed for a broader initial exploration of potential predictors before applying more stringent model selection criteria^39,40^. Multicollinearity among explanatory variables was assessed using Variance Inflation Factor (VIF) analysis^41^, which is essential for ensuring the stability and interpretability of regression coefficients^41^. The final model was selected using the backward elimination method, beginning with a full model and progressively removing nonsignificant variables at each step^39^.

## Results

### Data quality assessment and adjustment

**Fig. 1:**
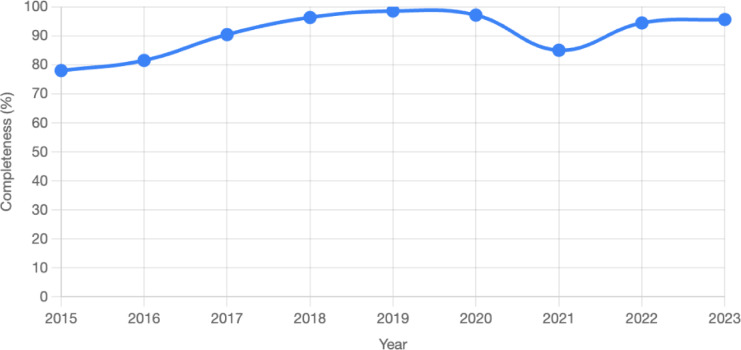
Data completeness trend for DTP-1, DTP-3 and facility deliveries (2015–2023).

Data collected from the DHIS2 database showed the same level of completeness in reporting across indicators, but with variation from 2015 to 2023, as shown in Fig. 1 below. While the overall trend shows an improvement in completeness, a slight dip in 2021 may reflect temporary reporting challenges during the COVID-19 pandemic.

A Kruskal-Wallis test was conducted to assess whether data completeness for DTP-1, DTP-3, and facility deliveries differed significantly across three time periods: before COVID-19 (2015–2019), during COVID-19 (2020–2021), and after COVID-19 (2022–2023). The median completeness values were 90.4, 91.05, and 95%, respectively. The test yielded a p-value of 0.135, indicating no statistically significant differences in data completeness across the three periods at a significance level of 0.05. This suggests that the reporting system for RI and maternal health indicators remained relatively stable despite disruptions caused by the COVID-19 pandemic.

Although there was no statistical difference in data reporting completeness across the pre-COVID, during-COVID, and post-COVID periods, the adjustment of coverage values using the predefined formula in the methodology was applied. This step enhanced the robustness of the analysis by accounting for subtle variations in completeness across years and indicators. The adjusted values presented in Table 2 provide a more reliable basis for the trend analysis and interpretation.

**Table 2:** Annual descriptive statistics for the dependent variables.

Year	Adjusted DTP-1 coverage (%) - mean	Adjusted DTP-1 coverage (%) - min	Adjusted DTP-1 coverage (%) - max	Adjusted DTP-3 coverage (%) - mean	Adjusted DTP-3 coverage (%) - min	Adjusted DTP-3 coverage (%) - max	Adjusted Proportion of facility deliveries - mean	Adjusted proportion of facility deliveries - min	Adjusted proportion of facility deliveries - max
2015	64.07	51.84	81.38	43.12	35.60	51.40	57.99	46.17	78.10
2016	93.70	63.58	116.48	69.14	48.10	90.60	44.58	28.20	61.05
2017	100.64	87.88	115.38	86.24	77.70	97.00	46.66	34.56	54.88
2018	82.68	73.16	92.03	74.10	69.30	79.90	49.49	41.94	55.83
2019	74.40	68.23	79.97	69.64	65.70	76.40	59.94	44.12	135.69
2020	83.09	65.87	108.99	77.41	63.10	98.10	67.33	53.96	109.92
2021	87.98	68.72	100.66	72.61	58.20	81.00	87.10	63.55	93.44
2022	85.73	77.37	103.09	75.84	69.30	82.60	95.76	87.89	105.77
2023	91.93	65.80	135.60	80.68	58.00	117.70	96.87	94.05	104.12

### Trends in routine immunisation and maternal health service access and utilisation (2015–2023)

**Fig. 2:**
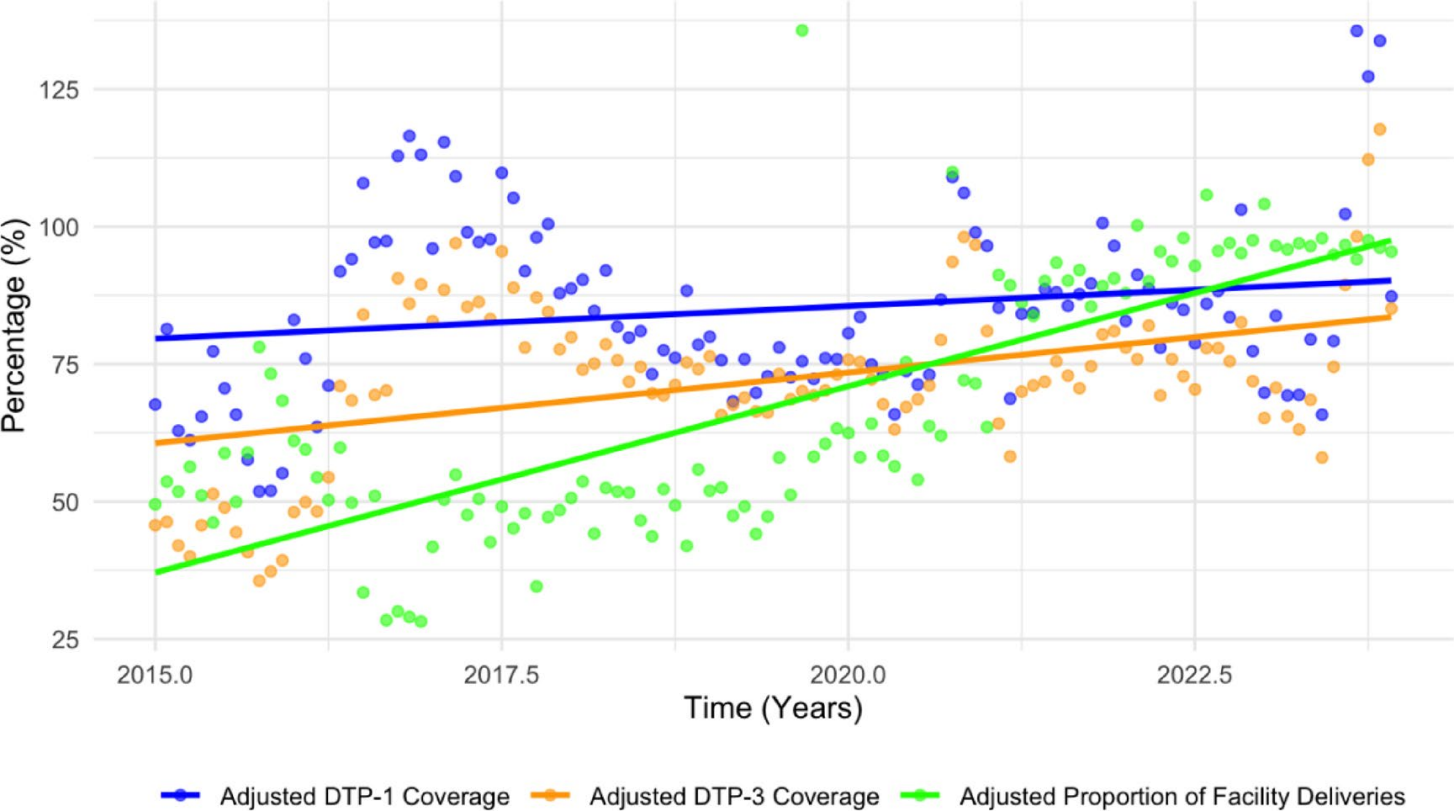
Trends in adjusted DTP-1, DTP-3 and facility delivery proportion (2015–2023).

Figure 2 presents the annual trends in adjusted coverage for DTP-1 and DTP-3 and the proportion of facility-based deliveries in Sokoto State from 2015 to 2023. The adjusted DTP-1 coverage increased steadily from 64.1% in 2015, peaking in 2017, followed by a slight dip in 2019, and a consistent improvement through 2023, reaching 91.9%. DTP-3 coverage followed a similar pattern, rising notably until 2018 and then declining in 2019–2020 before recovering strongly to 80.7% in 2023. These trends reflect progressive improvements in both access to and completion of RI services. The gap between DTP-1 and DTP-3 narrows over time, indicating better follow-up in immunisation schedules over time. The proportion of facility deliveries showed a pronounced increase, particularly during and after the COVID-19 pandemic. The coverage rose from 58.0% in 2015 to 96.9% in 2023, with a notable surge beginning in 2020.

### Temporal patterns in RI and facility-based deliveries

**Fig. 3:**
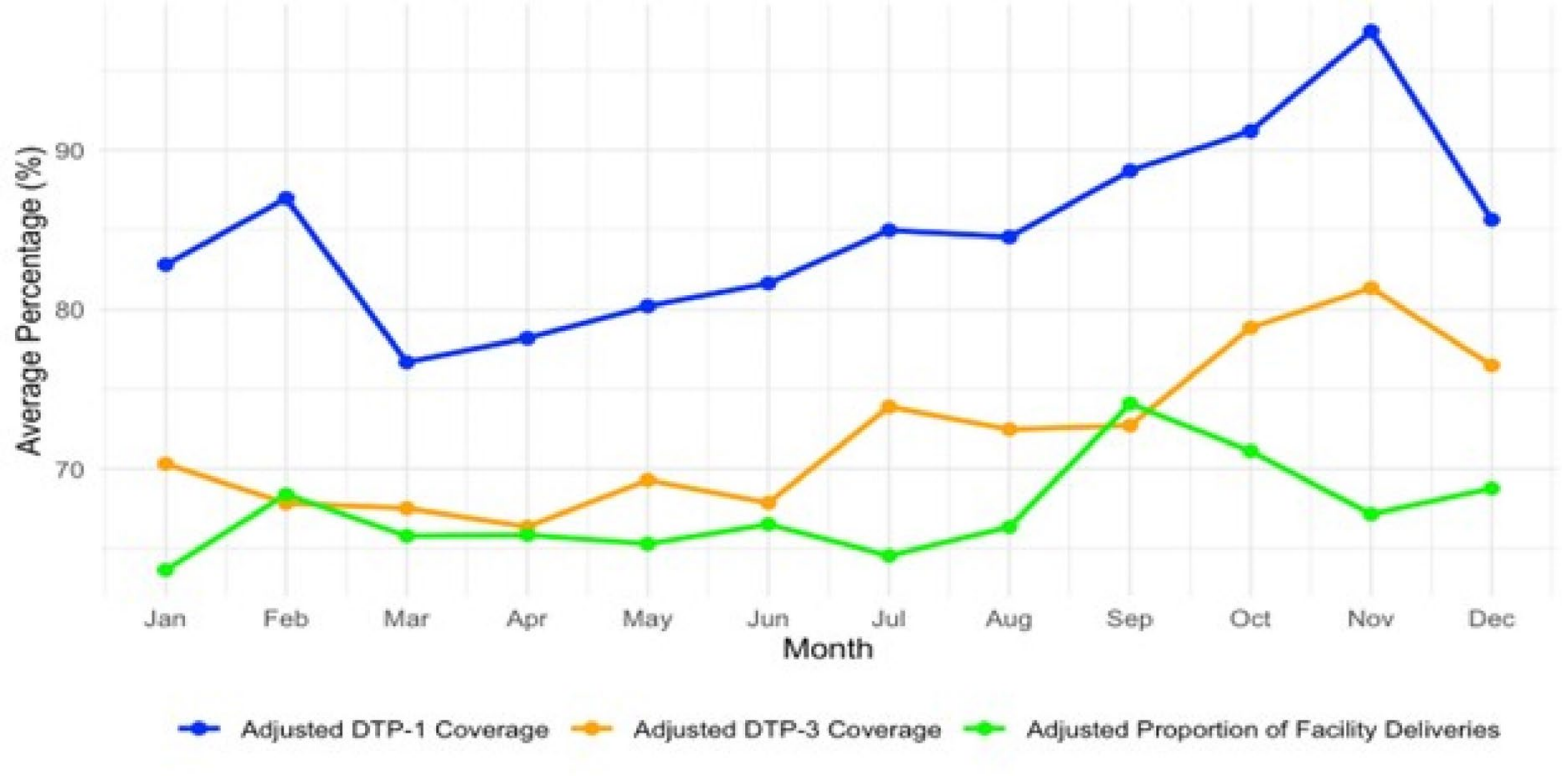
Monthly average trends in adjusted DTP-1, DTP-3 and facility delivery proportion (2015 to 2023).

Analysis of monthly average trends from 2015 to 2023 revealed distinct seasonal patterns in RI and maternal health service access and utilisation in Sokoto State, as shown in Fig. 3 below. The adjusted DTP-1 coverage increased progressively from 76.7% in March to a peak of 97.5% in November, while DTP-3 coverage increased from 66.4% in April to 81.3% in November, indicating intensified immunisation efforts toward the end of the year. The adjusted proportion of facility deliveries showed moderate fluctuation, with a low of 63.7% in January and a peak of 74.1% in September, suggesting seasonal influence. Further comparison between the first half of the year (January–June) and the second half (July–December) confirmed this pattern. The average adjusted DTP-1 coverage increased from 81.1% to 88.7%, DTP-3 coverage increased from 68.2% to 76.0%, and facility deliveries increased from 65.9% to 68.7% from the first half of the year to the second half.

### Impact of the COVID-19 period on RI and maternal health indicators

Bivariate analysis was conducted to examine the differences in adjusted RI and maternal health indicators between the COVID-19 and non-COVID-19 periods (Table 2). The mean adjusted DTP-1 coverage was slightly lower during the COVID-19 period (82.08%) than during the non-COVID period (85.23%), with a statistically significant difference (*p* = 0.032). Although the adjusted DTP-3 coverage also declined during the pandemic (75.68% vs. 78.46%), this difference was not statistically significant (*p* = 0.067). In contrast, the adjusted proportion of facility deliveries increased significantly during the COVID-19 period (72.21%) relative to the non-COVID period (66.34%), with a p-value of 0.019.

### Model diagnostics and multivariate regression analysis

Prior to model estimation, multicollinearity was assessed using a Variance Inflation Factor (VIF) analysis. All VIF values were below the conventional threshold of five, indicating no significant multicollinearity among the predictors (Table4). The highest VIFs were observed for the Annual Health Budget (3.12) and Annual Maternal Health Budget (3.25), suggesting a mild correlation, but not sufficient to warrant exclusion at the diagnostic stage. Both variables were retained in the initial models, although the Annual Maternal Health Budget was subsequently removed during backward elimination owing to non-significance (Table 5).

**Table 3:** Bivariate analysis of adjusted dependent variables by COVID-19 period.

Dependent variable	COVID-19 period	Mean (%)	Standard deviation (%)	Difference (yes - no)	*p*-value
Adjusted DTP-1 Coverage (%)	No	85.23	24.12	−3.15	0.032
	Yes	82.08	23.89		
Adjusted DTP-3 Coverage (%)	No	78.46	22.67	−2.78	0.067
	Yes	75.68	21.94		
Adjusted Proportion of Facility Deliveries (%)	No	66.34	29.45	5.87	0.019
	Yes	72.21	30.12		

**Table 4:** Variance inflation factor (VIF) for predictors.

Predictor	VIF
COVID-19 Period (Yes/No)	1.32
Minimum temperature	1.45
Thunderstorms (Yes/No)	1.28
Settlement type: urban (vs. Rural)	2.10
Settlement type: semi-urban (vs. Rural)	2.05
Annual health budget (NGN)	3.12
Annual maternal health budget (NGN)	3.25
Lagged DTP-1 coverage	1.67
Lagged DTP-3 coverage	1.65
Lagged proportion of facility deliveries	1.70

Dynamic time-series regression models were developed for each dependent variable, adjusted DTP-1 coverage, adjusted DTP-3 coverage, and adjusted proportion of facility deliveries using backward elimination to refine model parsimony. Each model began with a full set of potential predictors, including the COVID-19 period (yes/no), minimum temperature, Thunderstorms, settlement type (urban and semi-urban vs. rural), annual health and maternal health budgets, and lagged dependent variable. Predictors were removed iteratively based on their relative statistical insignificance, while the model fit was monitored using R-squared and F-statistics. Lagged dependent variables and fixed effects were retained throughout because of their theoretical relevance and consistent statistical significance (*p* < 0.001) (Table 5).

**Table 5:** Backward elimination steps for dynamic time series regression Models.

Dependent variable	Step	Model description	Removed predictor	*p*-value of removed predictor	*R*-squared	F-statistic (*p*-value)	Key changes
Adjusted DTP-1 coverage (%)	0	Full model: All predictors (COVID-19 Period, Min Temp, Thunderstorms, Settlement: Urban, Semi-urban, Health Budget, Maternal Budget, Lagged DTP-1)	-	-	0.69	46.81 (< 0.001)	Baseline model, all predictors included.
	1	Removed annual maternal health budget	Annual maternal health budget	0.312	0.68	46.50 (< 0.001)	R-squared slightly decreased, COVID-19 Period *p* = 0.029, no major changes.
	2	Removed minimum temperature	Minimum temperature	0.218	0.68	46.12 (< 0.001)	R-squared stable, COVID-19 Period *p* = 0.030, Health Budget *p* = 0.085.
		Final model: COVID-19 period, thunderstorms, settlement (urban, semi-urban), health budget, lagged DTP-1	-	-	0.68	46.12 (< 0.001)	Parsimonious model, COVID-19 Period significant (*p* = 0.030).
Adjusted DTP-3 coverage (%)	0	Full model: All predictors (COVID-19 period, min temp, thunderstorms, settlement: urban, semi-urban, health budget, maternal budget, Lagged DTP-3)	-	-	0.66	43.25 (< 0.001)	Baseline model, all predictors included.
	1	Removed annual maternal health budget	Annual maternal health budget	0.375	0.66	43.00 (< 0.001)	R-squared stable, COVID-19 Period *p* = 0.076, no major changes.
	2	Removed minimum temperature	Minimum temperature	0.310	0.66	42.89 (< 0.001)	R-squared unchanged, COVID-19 Period *p* = 0.078, Health Budget *p* = 0.123.
		Final model: COVID-19 period, thunderstorms, settlement (urban, semi-urban), health budget, lagged DTP-3	-	-	0.66	42.89 (< 0.001)	Parsimonious model, COVID-19 Period not significant (*p* = 0.078).
Adjusted proportion of facility deliveries (%)	0	Full model: All predictors (COVID-19 period, min temp, thunderstorms, settlement: urban, semi-urban, health budget, maternal budget, lagged facility deliveries)	-	-	0.73	49.12 (< 0.001)	Baseline model, all predictors included.
	1	Removed annual maternal health budget	Annual maternal health budget	0.184	0.73	48.90 (< 0.001)	R-squared stable, COVID-19 Period *p* = 0.020, Urban Settlement *p* = 0.030.
	2	Removed minimum temperature	Minimum temperature	0.238	0.73	49.45 (< 0.001)	R-squared unchanged, COVID-19 Period *p* = 0.019, Urban Settlement *p* = 0.027.
		Final Model: COVID-19 period, thunderstorms, settlement (urban, semi-urban), health budget, lagged facility deliveries	-	-	0.73	49.45 (< 0.001)	Parsimonious model, COVID-19 Period (*p* = 0.019) and Urban Settlement (*p* = 0.027) significant.

The final models demonstrated strong explanatory power, with R-squared values of 0.68, DTP-1 coverage, 0.66 for DTP-3 coverage, and 0.73 for facility deliveries. The COVID-19 period was significantly associated with a reduction in DTP-1 coverage (β = − 2.65, *p* = 0.030) and an increase in facility deliveries (β = 4.38, *p* = 0.019), but was not statistically significant for DTP-3 coverage (β = − 2.10, *p* = 0.078) (Table 6). Urban settlement type was positively associated with facility deliveries (β = 2.50, *p* = 0.027), whereas Thunderstorms and semi-urban settlement type did not reach statistical significance in any model. The annual health budget showed marginal significance for facility deliveries (*p* = 0.065), suggesting a potential resource effect on maternal health service utilisation (Table 6).

**Table 6:** Multivariate dynamic time series regression analysis (final models).

Variable	Adjusted DTP-1coverage (%)	Adjusted DTP-3 coverage (%)	Adjusted proportion of facility deliveries (%)
COVID-19 period (Yes vs. No)			
Coefficient	−2.65	−2.10	4.38
Standard error	1.21	1.18	1.87
p-value	0.030	0.078	0.019
Thunderstorms (Yes vs. No)			
Coefficient	0.90	0.79	1.21
Standard error	0.66	0.64	0.88
p-value	0.174	0.218	0.171
Settlement type: urban (vs. rural)			
Coefficient	−1.62	−1.36	2.50
Standard error	1.01	0.97	1.13
p-value	0.109	0.162	0.027
Settlement type: semi-urban (vs. rural)			
Coefficient	−0.85	−0.74	1.65
Standard error	0.78	0.76	0.93
p-value	0.275	0.331	0.077
Annual health budget (NGN)			
Coefficient	0.0004	0.0003	0.0005
Standard error	0.0002	0.0002	0.0003
p-value	0.085	0.123	0.065
Lagged dependent variable			
Coefficient	0.65	0.61	0.74
Standard error	0.08	0.07	0.09
p-value	0.000	0.000	0.000
Model fit			
R-squared	0.68	0.66	0.73
F-statistic	46.12	42.89	49.45
p-value (F-test)	< 0.001	< 0.001	< 0.001

## Discussion

This study presents a robust longitudinal analysis of RI and maternal health service access and utilisation in Sokoto State, Nigeria from 2015 to 2023. The findings revealed complex temporal dynamics, seasonal patterns, and multifactorial influences on service uptake, offering critical insights into health system performance in a high-burden resource-constrained setting. This analysis is particularly relevant in the context of persistent inequities in MNCH outcomes in northern Nigeria and the compounded disruptions introduced by the COVID-19 pandemic.

Despite widespread concerns about data system fragility during health emergencies, Sokoto State’s DHIS-2 reporting system demonstrated notable resilience. The Kruskal-Wallis test showed no statistically significant differences in data completeness across the pre-COVID (2015–2019), COVID (2020–2021), and post-COVID (2022–2023) periods. This stability may be attributed to early national preparedness efforts^42^. Although Sokoto’s first confirmed COVID-19 case was reported in April 2020, the Nigerian Centre for Disease Control (NCDC), Federal Ministry of Health, and development partners had already activated emergency response mechanisms nationwide by February 2020^42^. The repurposing of polio eradication infrastructure, particularly in northern Nigeria, may have further buffered Sokoto’s health system against reporting disruptions^43^.

### Annual trends in RI and maternal health service utilisation (2015–2023)

Adjusted DTP-1 and DTP-3 coverage increased steadily, peaking between 2017 and 2018, followed by a dip from 2019 to 2020 before recovering strongly to 91.9% and 80.7%, respectively, in 2023. The peak around 2017 corresponds to the intensive vaccination response to the large outbreak of Neisseria meningitidis Serogroup C in Nigeria from December 2016 to June 2017^44^. In addition, the trend likely reflects intensified RI campaigns and donor-supported initiatives following Nigeria’s polio-free certification, including the July 2017 round of the Sub-National Immunisation Days (IPDs) campaign in Sokoto State^45^. The peaks in 2017 and 2018 were followed by a decline in 2019, suggesting a possible decrease in available resources for intensified campaigns compared to the previous year. The coverage recovery from 2020, despite the pandemic, may be attributed to compensatory efforts, such as catch-up campaigns and integration of RI with COVID-19 response activities^42^. These trends reflect progressive improvements in both access to and completion of RI services. The gap between DTP-1 and DTP-3 narrows over time, indicating better follow-through in immunisation schedules over time. The DTP-3 rebound in 2022 indicates partial recovery, although the slower pace compared to DTP-1 underscores persistent gaps in the continuity of immunisation uptake after the first vaccine dose. Facility-based deliveries exhibited the most dramatic and consistent upward trend, with a sharp increase from 2020 (67.3%) coinciding with the pandemic response period. This counterintuitive increase may be explained by heightened risk perception, increased institutional trust, and emergency preparedness measures^46^.

### Seasonal and average monthly patterns

The monthly average trends across the nine-year period revealed distinct seasonal patterns. DTP-1 coverage peaked in November (97.5%), while DTP-3 reached its highest in the same month (81.3%), suggesting intensified immunisation efforts toward the end of the calendar year. These peaks may coincide with fiscal year-end targets, donor-reporting cycles, and seasonal campaigns. Facility deliveries showed moderate seasonal fluctuations, with the lowest in January (63.7%) and highest in September (74.1%). These patterns may be influenced by agricultural cycles, cultural practices, and climatic conditions, which affect access to health facilities. The second half of the year consistently showed higher averages across all the indicators, reinforcing the need for seasonally adaptive programming and resource allocation.

### Factors associated with access and utilisation of RI and maternal health services

Multivariate dynamic time-series regression models identified several key associations. The COVID-19 period was significantly associated with reduced DTP-1 coverage and increased facility deliveries, suggesting that, while immunisation outreach was disrupted, maternal health services may have benefited from heightened institutional engagement and risk awareness. However, the lack of statistical significance for DTP-3 coverage indicates that the completion of immunisation schedules remains vulnerable to systemic shock. The findings suggest that disruptions during the pandemic had a greater impact on initial access to immunisation (DTP-1) than on completion (DTP-3), likely because of interruptions in outreach and early contact services. The significant increase in facility deliveries during the pandemic (mean difference = + 5.87%, *p* = 0.019) is consistent with findings from other LMICs, where institutional delivery was perceived as safer owing to infection control measures and emergency preparedness^46^.

Settlement type emerged as a significant determinant of facility deliveries, with urban residence being positively associated with uptake. This finding aligns with UNICEF’s global data, which showed that in 2023, 93% of urban births were attended by skilled health personnel, compared to 77% in rural areas^47^. Geographic disparities in access to skilled care are well-documented in sub-Saharan Africa, where infrastructure, transportation, and health worker distribution are unevenly concentrated in urban centres^47^. The annual health budget was another variable which showed marginal significance for facility deliveries, suggesting that fiscal allocation may influence maternal health service utilisation. However, the absence of significance for RI indicators implies that budgetary increases must be accompanied by strategic programming, community engagement, and accountability mechanisms to translate into improved immunisation outcomes. Thunderstorm, which was not statistically significant, had positive coefficients across the models. This is consistent with the evidence that climatic events can disrupt access to health services, particularly in rural and underserved areas^48^.

The inclusion of lagged dependent variables, which were strongly significant across all models, underscored the path-dependent nature of health service utilisation in Sokoto State. This finding suggests that current levels of service uptake are shaped not only by immediate contextual factors, but also by the cumulative effects of prior service delivery efforts. In other words, past performance in immunisation and maternal health services appeared to influence future uptake, reinforcing the importance of continuity and consistency in health system interventions.

## Limitations

The use of secondary data, while pragmatic and resource-efficient, introduces inherent challenges related to data quality and completeness. Although adjustments were made to account for variations in reporting, the possibility of underreporting or inconsistencies, particularly in rural or hard-to-reach areas, remains a concern. Despite efforts to enhance the robustness through data validation and adjustment procedures, these limitations may have influenced the accuracy of some trend estimates.

The ecological design of the study, which relied on aggregated data at the LGA and state levels, restricted the ability to explore individual-level determinants of service access and utilisation. As a result, important behavioural, cultural, and socioeconomic factors that shaped health-seeking practices could not be examined. This limited the depth of interpretation, particularly in a setting as complex and heterogeneous as Sokoto State. Although the analysis incorporated a range of contextual variables, including climatic conditions, settlement type, and fiscal allocations, other potentially influential factors, such as health worker availability, security dynamics, and community trust in health systems, were not captured due to data constraints. These unmeasured confounders may have moderated the observed associations and should be considered in future studies.

The classification of the COVID-19 period as a binary exposure variable oversimplified the nuanced and evolving nature of the pandemic’s impact on health service delivery. Variations in lockdown intensity, level of acceptance of the COVID-19 vaccine, public health messaging, and community responses over time were not fully reflected in this approach. Moreover, although dynamic time-series regression models allow for the inclusion of lagged effects, the specification of lag structures is based on theoretical assumptions and may not fully capture the delayed or cumulative impacts of policy shifts, health campaigns, or environmental shocks.

## Conclusions

This study provides a comprehensive assessment of RI and maternal health service access and utilisation trends in Sokoto State over a nine-year period, offering critical insights into the temporal dynamics and contextual determinants of service uptake in a high-burden, resource-constrained setting. The findings revealed both progress and persistent challenges. Although coverage for DTP-1, DTP-3, and facility-based deliveries improved over time, particularly in the post-pandemic period, gaps remained in the continuity of immunisation and equitable access to maternal health services.

The analysis highlighted the complex interplay between health system resilience, contextual shocks such as the COVID-19 pandemic, and structural determinants including settlement type and fiscal allocations. The observed rebound in service utilisation following the initial pandemic disruption suggested a degree of adaptive capacity within the health system, yet the differential impact on DTP-1 and DTP-3 coverage pointed to vulnerabilities in sustaining follow-through immunisation services. The significant association between urban residence and facility deliveries further illustrates the entrenched geographic inequities that require targeted policies and programmatic interventions.

By leveraging dynamic time-series modelling and adjusting for key confounders, this study contributes robust empirical evidence to the discourse on health-system performance in fragile settings. The findings will not only inform the development of a pandemic-resilient framework for Sokoto State but also offer transferable lessons for similar contexts grappling with the dual burden of systemic underinvestment and external shocks.

## Data Availability

Data are available upon reasonable request to the corresponding author. Data can also be obtained through NIMET’s official website, and DHIS2 data through the Sokoto Primary Health Care Board.

## Abbreviations

ANC: Antenatal care
CBHI: community-based health insurance
COVID-19: Coronavirus disease 2019
DHIS-2: District health information system version 2
DTP: Diphtheria-tetanus-pertusis pertussis containing vaccines
HBM: Health behaviour model
MDG: Millenium development goals
MMEIG: Maternal moratility estimation inter-agency group
MMR: Maternal mortality rate
NBS: National bureau of statistics
NHIS: National health insurance scheme
NIMET: Nigeria meteorological agency
NMR: Neonatal mortality rate
PHC: Primary health care
PHIS: Private health insurance
PPE: Personal protective equipment
RIU: Routine immunisation utilisation
SDG: Sustainable development goals
SSA: Sub-saharan Africa
U5MR: Under-five mortality rate
UNICEF: United nations international children’s emergency fund
USAID: United States agency for international development
VPD: Vaccines preventable diseases
WHO: World Health Organization
